# Supplementary material for: Optimization of cryoprotectants and storage temperatures for preserving viability and probiotic properties of lyophilized bacterial strains from chicken gut
Source: PLoS One. 2025 Jul 10;20(7):e0328216. doi: 10.1371/journal.pone.0328216 (PMC12244651; doi:10.1371/journal.pone.0328216)
Supplement: Attachment — Submitted filename: Response to Reviewers.docx [file pone.0328216.s003.docx]

**Re: Resubmission of Manuscript (Manuscript No: PONE-D-25-19776) Entitled, "Optimization of lyophilization process for long-term storage of probiotic Bacillus and Staphylococcus: Effects on survival and functionality"**

Dear Editors and Reviewers,

Thank you for the opportunity to revise and resubmit our manuscript. We appreciate the time and effort the reviewers and the editorial team have invested in evaluating our work. We have carefully considered each reviewer's comments and made the necessary revisions to address their concerns. Below, we provide a point-by-point response to each of the comments.

**Additional Editor Comments:**

Due to use different Bacillus Spp., staphylococcus Spp. and one strain of Lactobacillus salivarius, indicate in the title in a correct way.

**Response:** Optimization of cryoprotectant formulations and storage temperatures for preserving viability and probiotic properties of lyophilized bacterial strains from chicken gut (L1-3)

**Reviewer #1:**

**Introduction:**

**Comment:** The manuscript requires improvement in the Introduction.

**Response:** We have thoroughly revised this section to enhance its clarity, logical flow, and relevance to the study. In particular, we have enhanced the background by including recent literature to better highlight the importance of the research topic, clearly identified the knowledge gap that our study aims to address and provided a more focused rationale for the study objectives.

**Comment:** What is the novelty of your study?

**Response:** The study employed a systematic evaluation of multiple cryoprotectant combinations across varying concentration gradients over an extended 12-month monitoring period—an approach that, to date, has not been thoroughly explored in probiotic preservation research.

**Material and Methods:**

**Comment:** Please add references for all protocols.

**Response:** All protocols used in this study were supported by appropriate references to ensure methodological validity and reproducibility (L125, L140, L155, L166, L188, L196, L209).

**Comment:** Add name and reference of the mediums and materials.

**Response:** The names and references of all media and materials used in this study have been clearly provided (L119, L123, L131, L134-136, L158, L168-173, L195, L197, L202, L212).

**Comment:** Antimicrobial activity section misses important data and assays may not have been conducted as recommended by antibiotic susceptibility test guidelines. According to the Clinical and Laboratory Standard Institute (CLSI) and European committee for antibiotic susceptibility testing (EUCAST) the determination of MICs with the method must use bacterial inoculum standardized at 5 x 10^5^ CFU/ml that does not seem to be the case here. Did you control the number of CFU/ml in your inoculum? Which OD cut-off was considered positive growth? If the antimicrobial testing was not performed according to guidelines, it must be discussed in the manuscript.

**Response:** Antimicrobial activity was evaluated using a modified version of the spot-inoculation method. The concentrations of both the test pathogens and probiotic inoculum were specified in terms of CFU/mL (L213-214). In this study, we considered OD_600_> 0.1 as indicative of positive growth.

**Comment:** What is test bacterial strain??

**Response:** The test bacterial strains were typically pathogenic bacteria. In this study, five pathogenic strains—*Escherichia coli, Yersinia enterocolitica, Vibrio cholerae, Klebsiella pneumoniae,* and *Salmonella typhi*—were used to evaluate the antimicrobial activity of the probiotic candidates.

**Results**
**Comment:** The figures 1, 2, and 3 are very illegible, the quality of the charts should be improved.

**Response:** We have enhanced the resolution and clarity of these figures to ensure they are fully legible. We have also adjusted the font sizes, color contrast, and line thickness where necessary to improve visual quality and readability.

**Comment:** I strongly advise authors to revise the explanation of the figures. Legends of many figures has to be increased.

**Response:** We have carefully revised the figure legends throughout the manuscript to provide more detailed and comprehensive explanations.

**Comment:** What does RD mean in the table’s legends?

**Response:** RD means resistance degree. It refers to what extent an organism's ability to withstand or counteract a particular factor, such as a disease, a chemical, or an environmental stressor.

**Discussion**
**Comment:**

-Some of the content presented here is repetition of the results.
-I feel that the Discussion is too long and can be shorted by 25% or so easily without losing important information.
-Some of the content presented here is subject to the Introduction.

-I also would recommend a better organization of ideas in the Discussion.

**Response:** We have revised the Discussion section accordingly. Redundant content overlapping with the Results and Introduction has been removed, and the length has been reduced as much as possible without compromising key information. We have also reorganized the section to improve clarity and logical flow of ideas. We believe these changes have strengthened the overall quality of the manuscript.

**Reviewer #2:**

**Comment:** Strengthen the introduction by highlighting the probiotic species of Bacillus and Staphylococcus.

**Response:** We have revised the Introduction by emphasizing the relevance of probiotic species within the genera *Bacillus* and *Staphylococcus*. Specifically, we now highlight their roles in promoting gut health, enhancing immune response, and improving nutrient absorption (L51-63).

**Comment:** Line 111, Bacterial cells washed twice with sterile distilled water. Won't washing bacteria with distilled water lead to their osmotic lysis?

**Response:** Yes, washing bacterial cells with sterile distilled water can lead to osmotic lysis. However, in this study, the risk of lysis was minimized by limiting exposure time and handling the cells gently as per manufacturer's protocol. Moreover, all the bacterial strains examined were Gram-positive, which are inherently more resistant due to their thick peptidoglycan cell walls.

**Comment:** In Table one, mention the used concentration of each compound in preparation of storage conditions.

**Response:** The concentration of each compound is provided in the table footnote (L152-153).

**Comment:** In Table1 (Suspended cells), what are the washed cells suspended in?

**Response:** The washed cells suspended in Phosphate Buffered Saline.

**Comment:** In 2.4 A 1 mL sample is mixed with 9 mL of SGF. Mention the CFU of bacteria in 1 mL of sample.

**Response:** CFU/mL has been included (L175).

**Comment:** According to 2.4, the text mentions that following gastric exposure, the surviving sample is transferred into 9 mL of SIF to replicate the intestinal phase. As the population of bacteria is important in their resistance rate, in related results, mention the population of survived bacteria for second test.

**Response:** CFU/mL has been included (L179).

**Comment:** Please include reference for each section of methods.

**Response:** All protocols used in this study were supported by appropriate references to ensure methodological validity and reproducibility (L125, L140, L155, L166, L188, L196, L209).

**Comment:** The effect of different excipient concentrations (2–10%) on the viability of various bacterial strains was not assessed in methods. The related results are presented in 3.1.

**Response:** Thank you for pointing this out. We acknowledge the omission and have now revised the Methods section to include a detailed description of how the effects of different excipient concentrations (2–10%) on the viability of the bacterial strains were assessed (Section 2.2).

**Comment:** In Line 202, how do you find Skim Milk Powder the most effective excipient for probiotic strains?

**Response:** We have revised the Results section to clearly state that Skim Milk Powder showed a steady improvement in bacterial viability, with optimal effects observed at a concentration of 7%.

**Comment:** For 3.6, authors need to substantiate pathogenic bacterial growth inhibition with plate photographs.

**Response:** Thank you for your valuable comment. We conducted the antimicrobial test over a 12-month period, evaluating 10 variants in triplicate during each trial. Given the extensive number of plates generated, including all images in the main manuscript was impractical. Therefore, we have summarized the data using inhibition zone ranges and provided detailed results in five supplementary tables.

We hope that current revisions are sufficient to make our manuscript suitable for publication in the PLOS ONE Journal and look forward to hearing from you at your earliest convenience.

Sincerely,
Adnan Habib

Research Associate

Animal Health Research Division

Bangladesh Livestock Research Institute

Dhaka-1341, Bangladesh
